# Sensor and Actuator Fault Diagnosis for Robot Joint Based on Deep CNN

**DOI:** 10.3390/e23060751

**Published:** 2021-06-15

**Authors:** Jinghui Pan, Lili Qu, Kaixiang Peng

**Affiliations:** 1 School of Automation and Electrical Engineering, University of Science and Technology Beijing, Beijing 100083, China; kaixiang@ustb.edu.cn; 2 School of Mechatronic Engineering and Automation, Foshan University, Foshan 528231, China; qulili@fosu.edu.cn

**Keywords:** fault diagnosis, sensor fault, actuator fault, deep convolutional neural network, robot joints

## Abstract

This paper proposes a data-driven method-based fault diagnosis method using the deep convolutional neural network (DCNN). The DCNN is used to deal with sensor and actuator faults of robot joints, such as gain error, offset error, and malfunction for both sensors and actuators, and different fault types are diagnosed using the trained neural network. In order to achieve the above goal, the fused data of sensors and actuators are used, where both types of fault are described in one formulation. Then, the deep convolutional neural network is applied to learn characteristic features from the merged data to try to find discriminative information for each kind of fault. After that, the fully connected layer does prediction work based on learned features. In order to verify the effectiveness of the proposed deep convolutional neural network model, different fault diagnosis methods including support vector machine (SVM), artificial neural network (ANN), conventional neural network (CNN) using the LeNet-5 method, and long-term memory network (LTMN) are investigated and compared with DCNN method. The results show that the DCNN fault diagnosis method can realize high fault recognition accuracy while needing less model training time.

## 1. Introduction

With the development of robot and control technology, various robots are widely used in industry. Different applications present specific requirements for robot systems, such as rapidity, robustness, and safety [1,2,3]. However, among all the indices required by applications, the controllability of the robot in fault state has become the most critical factor. Fault identification is the precondition for realizing this goal, which promotes the investigation of our research [4,5,6].

Robot systems cannot work without the support of different kinds of sensors and actuators. Miniaturization and multi-functionality are required for development. The rapid development of sensors, material science, and micro-electro-mechanical technology allows modern robot joint modules—such as hollow motor, servo driver, harmonic reducer, brake, and encoder—to be integrated within limited space [7]. Sensors and actuators are key components in the robot system, but their working environment is very complex, with electromagnetic interference, vibration, etc., which will affect the output of the sensors and then the actuators. Moreover, the variable load on manipulators is also a challenge for system state feedback or estimation. All of the above factors make the faults diagnosis of robot system sensors and actuators an urgent task [8].

In most robot system faults, sensor and actuator malfunction are the main causes of robot system failure. Therefore, diagnosis for the sensors and actuators is very important. In order to improve the reliability of robot joints and realize fault detection and fault-tolerant control of robot systems, researchers have been focused on fault detection and fault-tolerant control of robot joints for many years, and many practical fault diagnosis methods have been proposed. In [9], redundant sensors are used on the robot joint, and then fuzzy rules are designed to adjust the threshold of the fault signal adaptively to carry out fault diagnosis. In [10], for a six-degree-of-freedom robot joint system, low-cost MEMS magnetic, angular velocity, and gravity sensors are used to estimate the joint angle of a rotating manipulator. In [11], a discrete-time framework for fault diagnosis of robot joint sensors, force or torque sensors, and actuators is proposed. The redundant sensors are used on the robot joint, and the feedback values from redundant sensors and the estimated values calculated by two isolation observers are input into the fault decision system. The data from redundant sensors are used to provide information for a group of diagnostic observers to detect, isolate, and identify faults of joint actuators, force, or torque sensors.

However, there may be another consideration when using redundant sensors for fault diagnosis. A robot fault diagnosis system based on redundant sensors not only increases structural complexity, but also increases the hardware cost of the system. In addition, redundant sensors also increase the probability of a sensor fault when the running time of a robot system approaches the sensor’s life cycle.

In order to overcome the shortcomings of using redundant sensors for fault diagnosis, observers have been widely used. There are many novel theories that could be used to design state observers for robot fault diagnosis. A robot-fault diagnosis method using fuzzy logic is proposed in [12] to evaluate residuals. Fuzzy logic applied to robot fault diagnosis does not require any redundant sensors, but it relies on the fault model of the robot system. The sliding mode method can be seen everywhere in robot fault diagnosis. Daniele uses a second-order sliding mode observer for fault detection and isolation of the rigid manipulator of the COMAU robot and uses the suboptimal second-order sliding mode algorithm to design the input law of the proposed observer, which can detect a single fault on a specific brake or a specific sensor of the manipulator [13]. Since the high order sliding mode observer can detect possible faults in specific parts of the robot system, the sliding mode method is greatly expanded [14]. The observer design methods mentioned above are just some typical representatives, actually, there are many other methods that could be used for robot fault diagnosis, such as the output feedback method [15], nonlinear disturbance observer [16], and feedback linearization disturbance observer design method [17]. As it is well known, the difficulty of observer-based robot fault diagnosis lies in the gain design process [18].

Machine learning introduces an effective solution to the above problems caused by redundant sensors and observers. Typical application methods include, but are not limited to, genetic algorithm [19], support vector machine [20], cluster analysis [21], and neural network [22]. Among them, the neural network is widely used in the field of fault diagnosis because of its superior nonlinear fitting ability. Traditional methods of fault diagnosis manually realize feature extraction, so prior knowledge about fault information is needed, which increases the difficulty of analyzing the results. Neural networks, especially deep learning methods, can learn representations and patterns hierarchically from the input data, and realize effective feature extraction, so the deep learning method has the ability to model complex working conditions and output accurate predictions. Several typical deep learning methods have been successfully applied to fault diagnosis [23,24,25,26], including autoencoders [27], deep belief networks [28], and CNN [29]. The autoencoders and feature ensemble method is applied in actuator fault diagnosis [30]. Furthermore, the one-layer autoencoder-based neural network is proven to be effective in the task of fault classification [31]. The deep belief nets model is successfully applied for fault diagnosis in actuators using vibration signals [32]. One-dimensional CNN is used to analyze the raw time series data of the actuator and proved to be successful in diagnosing fault states [33], and a new CNN architecture based on LeNet-5 is set to process the bearing data set [34].

Considering that the output of sensor and actuator are similar when faults occur, the normal neural network fault diagnosis methods cannot exactly tell the difference between them. In this paper, the DCNN is used to diagnose sensor and actuator faults of robot joints. DCNN can extract the features from the input data and realize fault classification by increasing the depth of the network. In addition, flexible selection of convolution kernel width makes it an efficient way to deal with classification problems with weak characteristics. Actually, there may be many types of sensors and actuators; our research mainly focuses on the problems of fault diagnosis in position sensors for the robot joint and torque sensors for the actuator. The robot joint is forced to move in a sinusoidal trajectory with the control of actuator, and the position sensor feeds back corresponding signals under different sensor states. Position sensor and torque sensor are separately denoted by sensors and actuators in the following main text. The main contributions of this paper are as follows.

(1) This paper gives a fused sensor and actuator fault diagnosis model, where sensor and actuator fault could be expressed in one formulation. Still, different faults could be distinguished, which contributes to our study.
(2) This paper proposes a DCNN fault diagnosis method. There are several convolution blocks in the architecture, and the depth of each kernel on different blocks varies, which helps to extract features from the time domain of input data.
(3) Experiments with different neural network fault diagnosis methods, such as SVM, ANN, CNN, LTMN, are conducted and compared with DCNN to give a comparison.

The rest of this paper is organized as follows: Section 2 introduces the basic structure of DCNN, Section 3 introduces the neural network module training method based on deep CNN, simulation experiments are conducted in Section 4 and the results are compared, the authors conclude the paper at the end.

## 2. Basic Structure of DCNN

**Figure 1:**
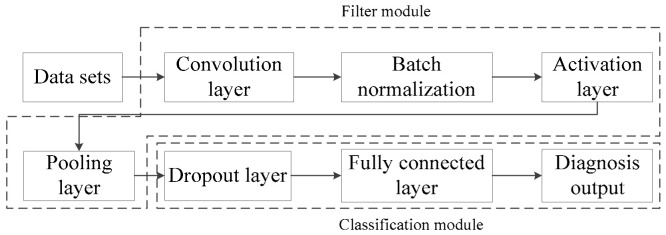
Diagram for CNN fault diagnosis system.

Generally, CNN consists of five parts: the convolutional layer, Batch normalization, activation layer, pooling layer, and dropout layer, as shown in Figure 1. In the CNN fault diagnosis architecture, each layer plays a different role. The following part of this section will briefly introduce the function of each layer.

### 2.1. Convolution Layer

**Figure 2:**
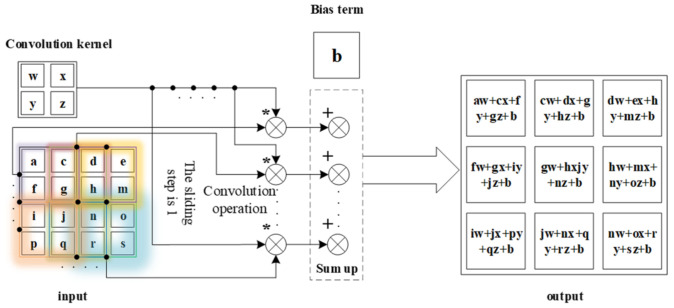
Diagram for convolution calculation.

The convolution layer is one of the most important parts of CNN. It is an effective feature extraction method using a convolution operation. The expression of convolution operation in discrete format is as follows.

(1) $$S(l,k)=X_{l}^{k}*W_{l}^{k}+b_{l}$$

where S(l,k) is the output of convolution core, $X_{l}^{k}$ is the input of convolution core, $W_{l}^{k}$ is the core function, and $b_{l}$ is the bias term. The numbers *l, k* are the serial number of the layer and convolution kernel. The mathematical operator ∗ in the above equation denotes the sum based on the multiplication of corresponding elements. Figure 2 gives an example of convolution operation with kernel dimension of 2 × 2. The input data with a dimension of 4 × 4 is divided into 9 subsets when the sliding step is 1, and each subset has the same dimension compared with the kernel. The multiplication of corresponding elements between kernel and subsets generates a 3 × 3 matrix, which is called the output of convolution core.

### 2.2. Batch Normalization

Batch normalization is one of the most computing-intensive layers in CNN architecture. Meanwhile, it is also a widely used method to accelerate and stabilize the CNN training process. The main purpose of batch normalization is to force the input data sets back to the standard normal distribution with the mean value of zero and the variance of one, so that the input of the nonlinear transformation function falls into the sensitive region, which could avoid gradient loss.

The input of batch normalization comes from the output of the convolution core, and the output $y_{i}$ of batch normalization is relative to data features, as shown in the following equation:

(2) $$y_{i}=\gamma {\hat{x}}_{i}+\beta$$

where the gain γ and the bias term β are used to accelerate the convergence process. ${\hat{x}}_{i}$ is the function of mean value and standard deviation $\sigma_{\beta }$, see the following equation.

(3) $${\hat{x}}_{i}=\frac{1}{\sigma_{\beta }}(x_{i}-u_{\beta })$$

(4) $$\left\{\begin{array}{l} \sigma_{\beta }=\sqrt{\frac{1}{m}\sum_{i=1}^{m}{\left(x_{i}-u_{\beta }\right)}^{2}+\varepsilon }\\ u_{\beta }=\frac{1}{m}\sum_{i=1}^{m}x_{i} \end{array}\right.$$

where ε is a very small positive value, $x_{i}$ is the output of convolution operation module, *m* is the length of data sets.

### 2.3. Activation Layers

A convolutional neural network consists of stacked layers, with two basic parts on each layer; separately, they are trainable linear convolutional filters and a fixed nonlinear activation function. The activation function used in our research is ReLU [35], and its expression is as follows.

(5) $$g(t)=\max(t,0)=\left\{\begin{array}{cc} 0, & t<0\\ t, & t>0 \end{array}\right.$$

**Figure 3:**
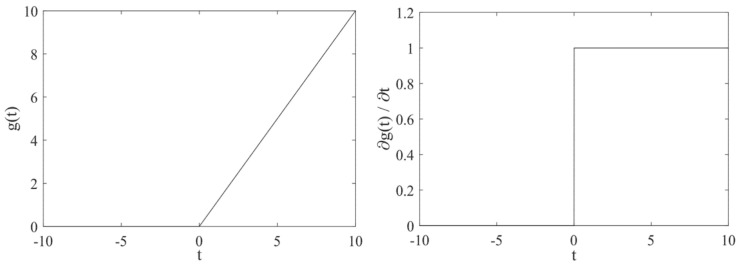
Graph of ReLU.

The ReLU function indicated by Equation (5) is a nonlinear function, where its derivative is one when *t* > 0, and zero when *t* ≤ 0. The graph of the ReLU function is shown in Figure 3. The use of the ReLU function eliminates the problem of gradient vanishing compared with Sigmoid and tanh functions.

### 2.4. Pooling Layer

**Figure 4:**
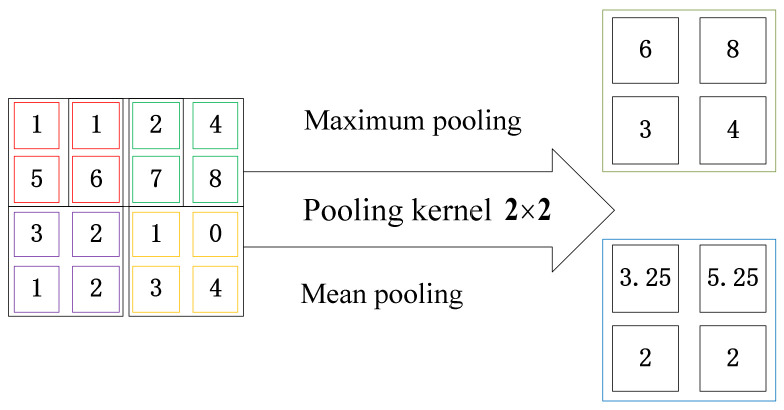
Operation of pooling process.

The pooling layer is used to reduce the amount of feature data needed and enhance the operational performance of the network. The main pooling methods could be classified into two categories, and they are maximum value pooling and mean value pooling methods. Figure 4 shows the basic operation of the above two pooling methods, where the input data dimension is 4 × 4, and the pooling kernel dimension is 2 × 2. The pooling kernel matrix is used to multiply with input data using the sliding step of 2. The maximum value pooling method chooses the maximum value in all four data with the same color, while the mean value pooling methods get the average value.

### 2.5. Dropout Layer

**Figure 5:**
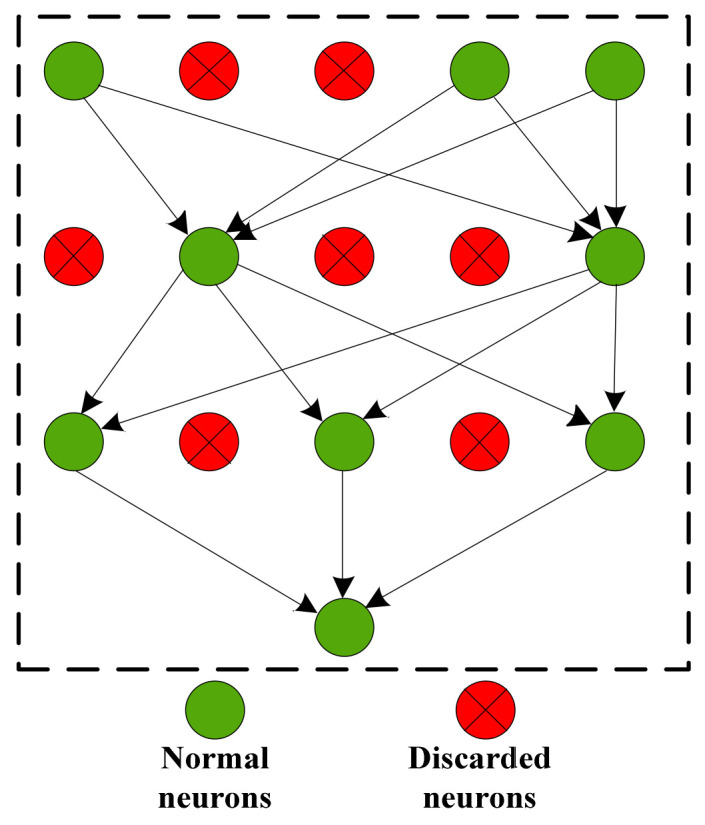
Process of dropout.

To alleviate the overfitting problem of the neural network, some neurons in the hidden layer are temporarily discarded according to a certain proportion in the training process of the neural network, and all neurons are restored when used again. When the rate of discard is fifty percent, each neuron has one half of the probability to be removed, so that the training of one neuron does not rely on another one, thus, the interaction between features is weakened, so as to alleviate the overfitting phenomenon caused by severe neural network depth [36]. The dropout process is shown in Figure 5.

### 2.6. Fully Connected Layer

**Figure 6:**
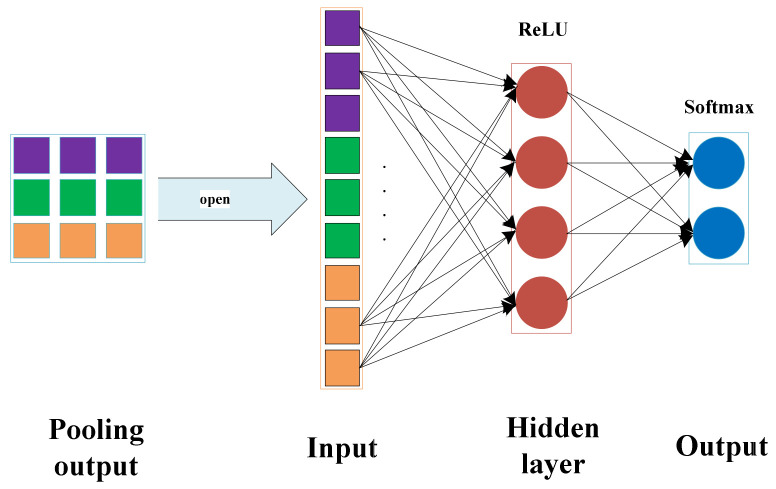
Full connection process.

The fully connected layer is a classifier, which can map the features extracted from the filter modules to the marked data features [37]. The input of the fully connected layer is the output of the last pooling layer. Each neuron of the fully connected layer relates to all other input neurons. Then the data on each neuron is processed using the ReLU activation function, and the final output is classified through the Softmax regression method. Taking one dimensional convolution as an example, the full connection process is shown in Figure 6.

### 2.7. Loss Function

There should be an evaluation method for the precision of the model during the training process. In order to make the prediction outputs of the neural network model consistent with the real values, cross-entropy function is calculated to evaluate the differences between them. The cross-entropy function is called loss function and its expression is as follows.

(6) $$loss\_ce=-\sum_{n}P(y)\log P(\hat{y})$$

where *y* denotes the known result, $\hat{y}$ is the output of the network, *P*(*y*) is the probability distribution of known results, *P*($\hat{y}$) is the probability distribution of network prediction results, and *loss_ce* is the cross-entropy function. The cross-entropy function emphasizes the difference in the probability distribution of each data class and is often used in the multi-classification problem of neural networks.

**Figure 7:**
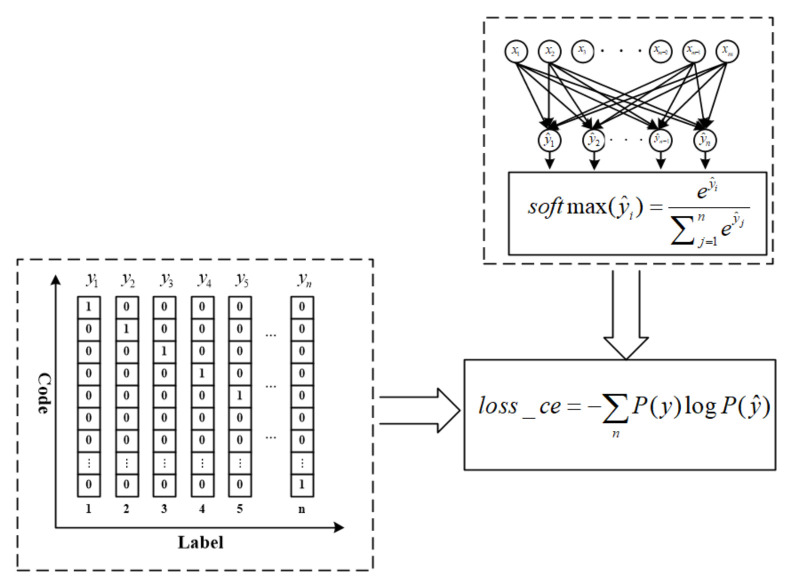
Calculation of loss function.

The softmax regression method makes the outputs of the neural network submit to established distribution. Assuming outputs are divided into *n* classes, then ($\hat{y}$_1_, $\hat{y}$_2_,…, $\hat{y}$*_n_*) is obtained. When the Softmax function is used, the outputs of the neural network meet the desired probability distribution. The calculation of the loss function is shown in Figure 7.

(7) $$Soft\max({\hat{y}}_{i})=\frac{e^{{\hat{y}}_{i}}}{\sum_{j=1}^{n}e^{{\hat{y}}_{j}}}$$

(8) $$\forall \hat{y}\;P(\hat{Y}=\hat{y})\in \left[0,1\right]\;\cap \;\sum P(\hat{Y}=\hat{y})=1$$

## 3. Sensor and Actuator Fault Diagnosis Framework Using DCNN

**Figure 8:**
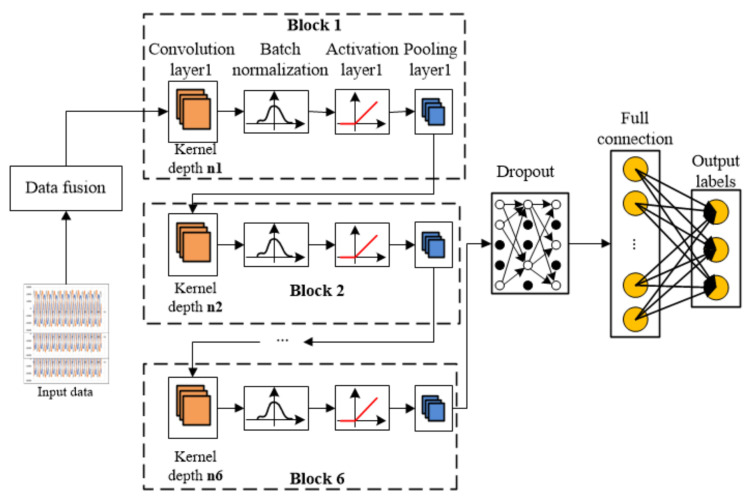
Fault diagnosis framework of DCNN.

The proposed robot joint sensor and actuator fault diagnosis framework based on DCNN is shown in Figure 8. It is shown that the whole fault diagnosis framework can be divided into data fusion, multi-level feature extraction, dropout of neurons, full connection, and diagnosis output parts. There are six “Blocks” in the framework and each framework consists of the convolution layer, batch standardization layer, activation layer, and pooling layer.

The convolution layer parameters of Block1 are [32 * 1, 8, 4], where 32 * 1 represents the dimension of one-dimensional convolution kernel, convolution depth is 8, and sliding step is 4. The convolution parameters of Block2 to Block6 are [3 * 1, 16, 1], [3 * 1, 32, 1], [3 * 1, 64, 1], [3 * 1128, 1], [3 * 1128, 1]. The pooling parameters of Block1 are [2 * 1, 8, 2], where 2 * 1 represents the dimension of the pooling kernel, pooling depth is 8, and sliding step is 2. The pooled layer parameters from Block2 to Block6 are [2 * 1, 16, 2], [2 * 1, 32, 2], [2 * 1, 64, 2], [2 * 1128, 2], [2 * 1, 16, 2]. The pooled output of Block6 is used as the input of Dropout, and the rejection rate is set to 50% to slow down the over fitting of the model. The number of neurons in the full junction layer is 100, and they are connected to 10 categories.

### 3.1. Data Fusion

This paper aims at the fault diagnosis problem of robot sensor and actuator, so there are many kinds of input data that contain a variety of fault information. Therefore, this paper adopts a data fusion scheme to unify the sensor fault and actuator fault in one expression, so that the output of the fused model contains both sensor and actuator fault characteristics. The first thing to do is to establish the mathematical model of sensor and actuator respectively. According to the laws of mechanics, the mathematical model of the actuator is as follows.

(9) $$S_{1}:\left\{\begin{array}{l} \dot{x}=Ax+Bf(q,\dot{q},\tau )\\ y=Ex \end{array}\right.$$

where,

$A=\left[\begin{array}{cc} 0 & 0\\ 0 & 1 \end{array}\right],B=\left[\begin{array}{c} 0\\ 1 \end{array}\right],E=\left[\begin{array}{cc} 1 & 0\\ 0 & 1 \end{array}\right]$, $f(q,\dot{q},\tau )=D^{-1}(q)[\tau -C(q,\dot{q})\dot{q}-G(q)]$, $x={\left[x_{1}\;x_{2}\right]}^{T}={\left[q\;\dot{q}\right]}^{T}$, and $q,\dot{q}\in R^{n}$ are state variables of angular position and angular velocity.

From Equation (9), the torque equation of the actuator can be obtained, as follows.

(10) $$D(q)\ddot{q}+C(q,\dot{q})\dot{q}+G(q)=\rho \tau +f_{a}$$

where τ is the vector for torque with a dimension of *n*; *D*(*q*) and $C(q,\dot{q})$ are square matrices with the dimension of *n*, denoting inertia matrix and Coriolis force matrix, respectively; and $G(q)\in R^{n}$ is the gravity moment vector. *ρ* ∊ [0,1] is the effective factor of the actuator. *ρ* = 0 means the actuator is completely broken. *f_a_* is the bias term and its value is positively correlated with the degree of actuator damage. The actuator faults with different combinations of *ρ* and *f_a_* are listed in Table 1.

It could be seen from Equation (9) that the output *y* variable of the system *S*_1_ is the state variable *x* multiplied by the coefficient matrix *E*. Thus, the robot sensor fault can be directly expressed by the output equation, as follows.

(11) $$S_{2}:\left\{\begin{array}{l} \dot{x}=Ax+Bf(q,\dot{q},\tau )\\ y=\lambda Ex+f_{b} \end{array}\right.$$

where *λ* ∊ [0,1] is the effective factor of the sensor. *λ* = 0 means the sensor does not work anymore. *f_b_* is the bias term and is positively correlated with the degree of sensor damage. The sensor faults with different combinations of *λ* and *f_b_* are listed in Table 2.

From the robot joint sensor fault model and actuator fault model, it could be seen that both of them affect the system in a different way. However, through the model derivation and transformation, the sensor fault can be transformed into an actuator fault through the first-order filter [38], which simplifies the model. The sensor and actuator fault data are fused according to the following formula.

(12) Fault=aΔSensor+bActuator

where *Fault* denotes fault data set needed, Δ*Sensor* represents the difference between sensor output and settings, *Actuator* is the output of actuator, *a* and *b* are sensor and actuator faults coefficient respectively. Equation (12) has now unified two kinds of fault from sensor and actuator, which helps to obtain training data sets.

The robot sensor and actuator mixed faults studied in this paper are listed in Table 3. F1 to F10 will be used to represent different fault labels in the following description for ease of use.

### 3.2. Training of Model and Diagnosis

The fault diagnosis model needs to be well trained before it is used to realize fault diagnosis. The basic training process of the fault diagnosis model based on the DCNN proposed in this paper is as follows.

Fused data containing single or mixed fault of sensor and actuator is input into Block1 (refer to Figure 8). Convolution layer1 uses kernel1 to carry out convolution operations. The result of the convolution operation is input into the batch standardization module, and the extracted data features are standardized to make the extracted feature data conform to the standard normal distribution. The activation function is then used to activate the neurons. The activation function used here is the ReLU function, and it owns the fine linear property, which overcomes the saturation effect of using Sigmoid or Tanh functions.

Finally, the activated features are input into the pooling layer. The pooling method used here is the maximum pooling method, which can extract the maximum features. The above operation completes the main steps of Block1. The output of Block1 is propagated to Block2, and the above steps conducted in Block1 are repeated until all six Blocks finished the corresponding operation.

After feature extraction is finished, the data should flow into the fully connected layer. however, in order to speed up the training process, the dropout layer is introduced to inactivate some neurons with a certain probability, and then the rest of the neurons come into the fully connected layer, and finally the fault diagnosis model gives the prediction labels.

**Figure 9:**
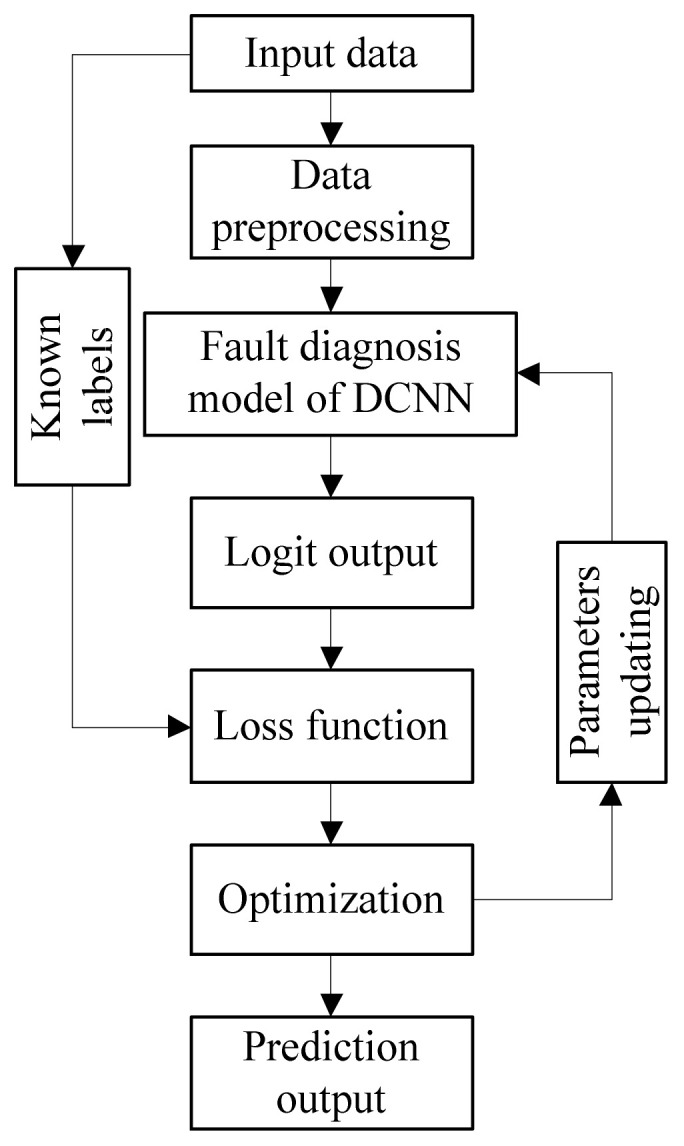
Process of training based on DCNN.

In the training stage of the model, updating the network weight parameters is key. Figure 9 gives the flowchart of the parameters updating process, where the network outputs are evaluated using the loss function to determine the direction of parameter update. After the model is well trained, the real-time data from the robot sensor and actuator can be propagated into the fault diagnosis model to realize fault diagnosis.

## 4. Experiment and Analysis

Data needed is based on multi-joint cooperative robot AUBO i3, with a DOF of 6, and six revolute joints with a maximum working radius of 625 mm. Our Matlab model is constructed based on the above platform. There are several position sensors and one actuator for each joint, so it is necessary to monitor their working state. Experiments using different fault diagnosis methods are conducted to reveal the effectiveness of the proposed DCNN. A PC with i7-10510U 1.8Ghz processor and 16G of RAM is used. The PyCharm software is installed, and combined with a Python3.6 interpreter. All the algorithms are implemented on Keras with Tensorflow as its backend.

**Figure 10:**
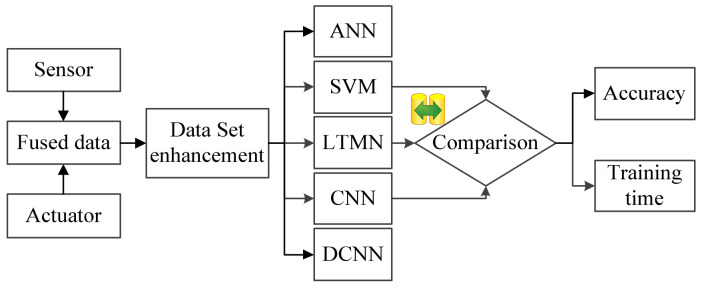
The basic architecture of the Comparative experiments.

The basic architecture of several experiments is shown in Figure 10, where the fused data is expanded through the data set enhancement method, and then several neural network fault diagnosis methods are investigated and their results are compared.

### 4.1. Data Sets Enhancement

**Figure 11:**
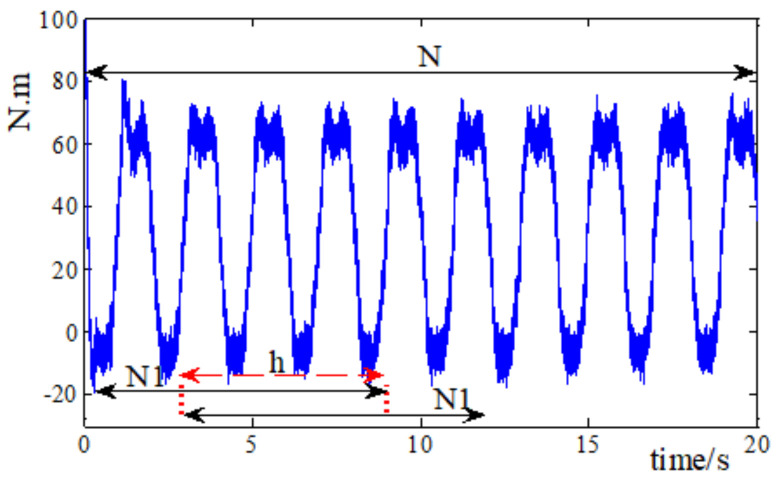
Diagram of data set enhancement.

Considering that the amount of fault sample is not enough in a real robot joint system, in this paper, the data set enhancement method is used to expand the fault sample data sets and improve the generalization ability of the model. In order to expand the acquired robot joint fault data, a sliding sampling data set enhancement method is proposed in this paper, and the schematic diagram of data set enhancement is shown in Figure 11.

The system data over a period is obtained, and a sample data segment with *N*_1_ points is needed for single training. Assuming that the length of the data obtained is *N*, then the network could be trained by *N*/*N*_1_ times according to the above method. In order to expand the coefficient of utilization for data, the start point of the second data set is *h* backwards compared to the first one, and the rest is roughly the same. The difference of samples before and after the data set enhancement method is used is shown in the following equation.

(13) $$\frac{N-h-1}{N_{1}-h-1}-\frac{N}{N_{1}}=\frac{N-N_{1}}{N_{1}\left(N_{1}-h-1\right)}\geq 0$$

It is obvious that when the sliding step size is small, which means *h* is quite large, more data samples could be obtained, which can well meet the needs of data sets in the training process. Here in our research, the sliding step selected is *h* = 29, and thus we could obtain plenty of data samples for model training and validation.

**Figure 12:**
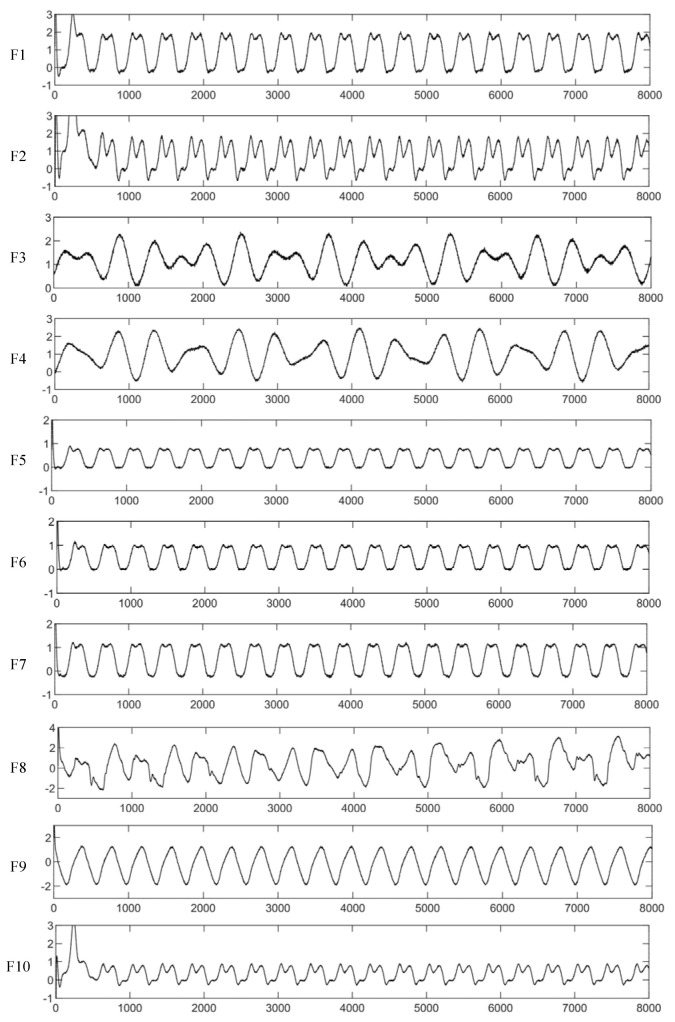
Data samples under different working conditions.

We have constructed a robot fault model in MATLAB/Simulink according to Equation (12), and the fault data needed in our study is obtained. In the process of data acquisition, the sampling rate is set to1000 hertz, and the sampling time is 8 s, so 8000 sample points for each kind of conditions are obtained, see Figure 12. The aforementioned data set enhancement method is adopted, so we can get 2000 samples for each kind of conditions. The 2000 samples are divided into three parts, and they are training, verification, and testing samples respectively, and the proportion of the above samples are 70%, 20%, and 10%, which would be used in the later model training and verification process. It should be noted that the input data set is one-dimensional, which is different from the conventional two-dimensional image data.

From Figure 12, it is very clear to see that some of the fault types could be easily distinguished, such as F1, F3; F2, F9. while some of them could not, such as F3, F4. When the “F3” fault happens, if the diagnosis model output is “F4”, it may bring adverse effects. Thus, realizing high accuracy prediction makes sense.

### 4.2. Hyper Parameters of DCNN

Hyper parameters of the neural network include learning rate, regularization parameters, and iterations. Actually, these hyper parameters control the values of the weight coefficient. According to existing researches, hyper parameters in deep learning algorithms not only affect the performance of the algorithm itself, but also affect the expression ability of the trained model. This paper takes the advice of Bengio [39]. The corresponding hyper parameters are set according to whether these hyper parameters can increase or decrease the capacity of the model.

In the process of parameter updating, the exponential decay learning rate is adopted. At first, a large learning rate is used to get the optimal solution quickly, and then the learning rate is gradually reduced to keep the model stable in the later stage of training. The initial learning rate $\eta_{0}$ is set to 0.2, and the decay learning rate ξ is set to 0.99, so the decay rate is updated per round. The expression for decay rate is as follows.

(14) $$\left\{\begin{array}{l} \eta =\eta_{0}\times \xi^{\left(H/L\right)}\\ H=Epoch/Batch\_k \end{array}\right.$$

where η denotes exponential decay learning rate, *H* stands for the number of the current round, and *L* represents the turns the decay should be executed once, *Batch_k* is the number of iterations. When a complete data set passes through the neural network once and then returns, this process is called *Epoch*.

In order to alleviate the overfitting of the neural network, the *l*_2_ regularization method is used in this paper. Regularization is to introduce the model complexity index into the loss function and suppress the noise in the training data set by weighting the parameters in the neural network. The expression of the loss function is as follows.

(15) $$\left\{\begin{array}{l} Loss=Loss\_all+REGULARIZER\times \\ \;\;\;Loss(w)\\ Loss(w)=\sum_{i}\left|w_{i}\right| \end{array}\right.$$

where *Loss_all* represents the loss function of all parameters in the model, *REGULARIZER* is the regularization weight, *w* generally refers to the weight in the forward propagation of neural network, and *Loss(w)* is the result of $l_{2}$ regularization of parameter *w*.

The Adma optimal algorithm is used [40] and the procedure of weight updating is as follows.

Step 1: give the iteration step ε=0.001.

Step 2: set the decay rate for matrix calculation, $\rho_{1}=0.99,\rho_{2}=0.999$.

Step 3: Determine the convergence threshold $\delta =10^{-8}$.

Step 4: Initialize network weight θ, 1st and 2st moment variables s,r, and set s=0,r=0.

Step 5: Set the simulation time step to 0.0001.

Step 6: Small data set with *m* samples are collected from the training set, using {*x*^(1)^,*x*^(2)^,…,*x*^(m)^}. to denote it, and set corresponding goals $y^{\left(i\right)}$.

Step 7: Calculate gradient variable $g\leftarrow \frac{1}{m}\nabla_{\theta }\sum_{i}L(f(x^{\left(i\right)};\theta ),y^{\left(i\right)})$, and update biased first moment estimation $s\leftarrow \rho_{1}s+(1-\rho_{1})g$ as well as biased second moment estimation $r\leftarrow \rho_{2}r+(1-\rho_{2})g$.

Step 8: Correct the deviation of the first moment $\hat{s}\leftarrow \frac{s}{1-\rho_{1}^{t}}$ and deviation of the second moment $\hat{r}\leftarrow \frac{r}{1-\rho_{2}^{t}}$.

Step 9: Calculate incremental weight error $\Delta \theta =-\varepsilon \frac{\hat{s}}{\sqrt{\hat{r}}+\delta }$, and update it to the network weight θ←θ+Δθ.

Step 10: If the convergence threshold in Step 3 is not met, then, back to Step 6, otherwise end the iterative process.

### 4.3. Simulation and Results

**Figure 13:**
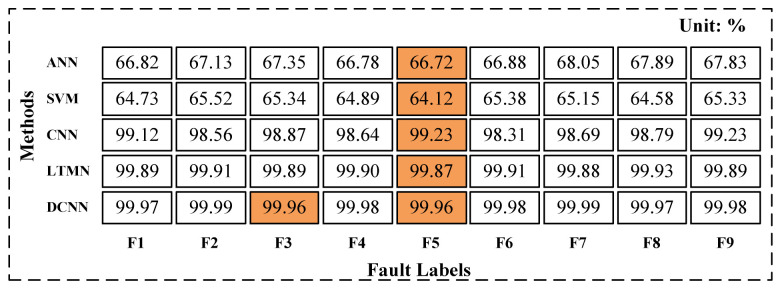
Fault diagnosis accuracy on training process of the model.

In order to verify the feasibility and effectiveness of the DCNN used in this paper for robot joint sensor and actuator fault diagnosis, the ANN, SVM, CNN, and LTMN methods are studied for comparative analysis and verification. The diagnosis accuracy of the different network is shown in Figure 13.

As shown in Table 3, There are nine fault states for sensor and actuator. For each fault state F1 to F9, 2000 samples are obtained and then divided into training, testing, and verification data subset. The accuracy of different diagnosis methods is summarized in Figure 13. It can be seen that the accuracy of fault recognition using DCNN is significantly improved compared with ANN, SVM. The average accuracy of the DCNN in the training process is over 99%. However, it is worth noting that the lowest accuracy of the five methods appears in F5. It could be interpreted that F5 is a mixed fault of sensor and actuator, and Figure 12 shows that there is no obvious difference between the fault curves of sensor and actuator, so the fault diagnosis models cannot effectively tell the difference between them.

**Figure 14:**
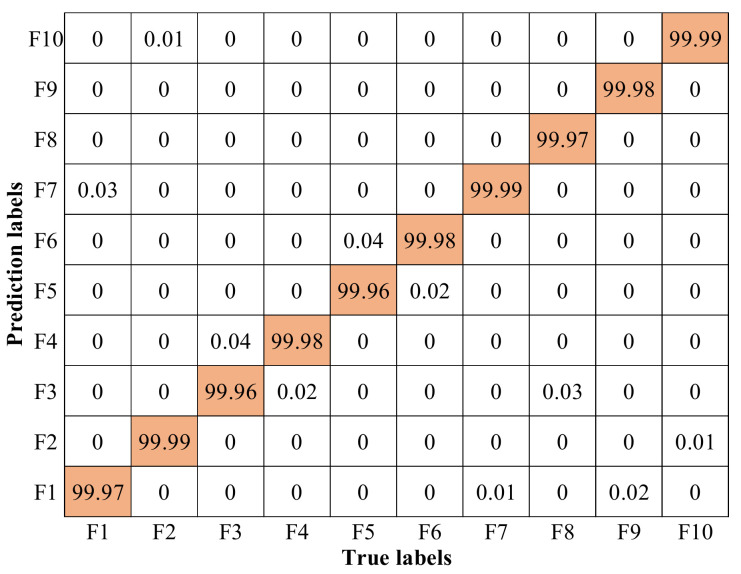
Confusion matrix of DCNN.

The confusion matrix of DCNN used on robot sensor and actuator fault diagnosis is shown in Figure 14. It could be seen that none of the fault types could be 100% recognized, and meanwhile, confusion matrix data shows that between that “Misjudgment” categories, their waveforms seem alike.

Further experiment research is conducted to compare the fault diagnosis effect of CNN, LTMN, and DCNN on robot joint sensor and actuator faults, and the accuracy and loss function figure of each diagnosis method in the training set and testing set are drawn with the help of TensorFlow.

**Figure 15:**
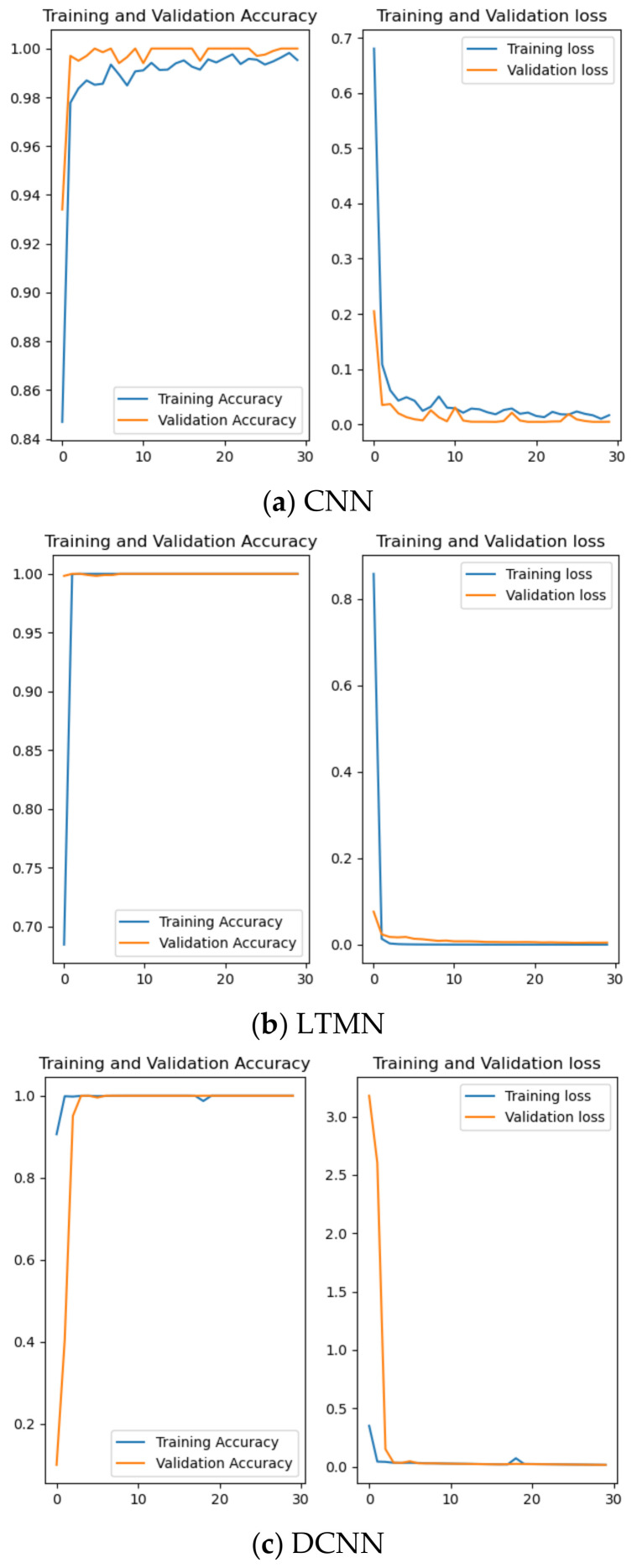
Accuracy and loss of three kinds of neural networks.

Three of five fault diagnosis methods with an accuracy over 90% are investigated. From Figure 15, it can be seen that the LTMN and DCNN could realize a fault recognition rate of 100% on both training and testing data sets, and there is no gap between training and testing loss function, which shows the robustness of LTMN and DCNN.

**Figure 16:**
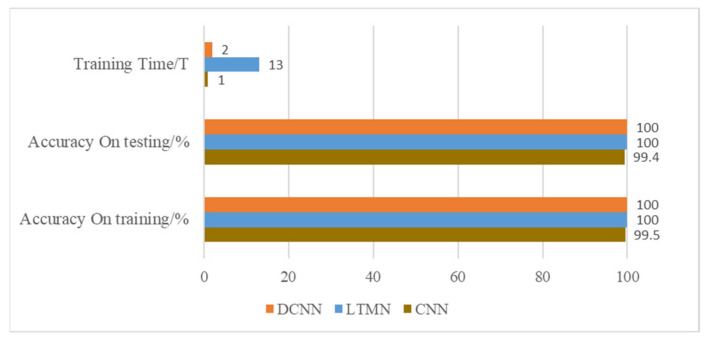
Accuracy and training time of three kinds of neural networks.

The model training time and accuracy are shown in Figure 16. This shows DCNN needs fewer training time but still gets the maximum diagnosis accuracy, which proves the high performance of the proposed DCNN fault diagnosis method.

**Figure 17:**
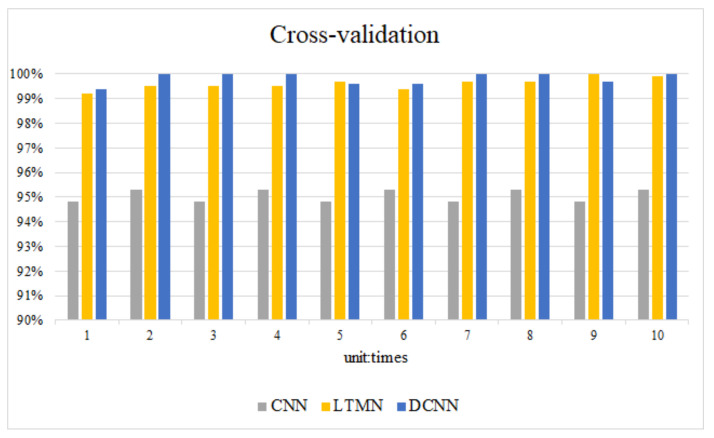
Cross-validation result.

The initial value of the neural network is randomly given, in order to eliminate the influence of accidental factors. This paper uses the cross method to train the model 10 times, and each time the network is initialized with a random value. The accuracy of CNN, LTMN, and DCNN are shown in Figure 17.

As can be seen from Figure 17, the recognition accuracy of DCNN in each experiment is more than 99%, and its lowest accuracy is 99.6%. Thus, the model initial value has little effect on fault diagnosis accuracy, which also proves the robustness of DCNN.

## 5. Conclusions

The DCNN fault diagnosis method is used to recognize the sensor and actuator faults of the robot system. The robot sensor and actuator output data are fused. In order to increase the number of training samples, the fault data set is expanded way of the data set enhancement method, and then the fault diagnosis is carried out using a deep convolution neural network. SVM, ANN, CNN, and LTMN-based neural network fault diagnosis methods are compared with the proposed DCNN and conclusions can be drawn that DCNN can better extract the fault information from the original input data and makes a more accurate classification of the sensor and actuator fault types.

## Figures and Tables

**Table 1:** Actuator fault type.

*ρ*	*f_a_*	Fault Type
1	Not zero	Constant deviation fault
0 < *ρ* < 1	0	Constant gain fault
0	Not zero	Actuator stuck
0	0	Actuator broken

**Table 2:** Sensor fault type.

*λ*	*f_b_*	Fault Type
1	Not zero	Constant deviation fault
0 < *λ* < 1	0	Constant gain fault
0	Not zero	sensor stuck
0	0	sensor broken

**Table 3:** Investigated Sensor and actuator fault type.

Label	Fault Description
F1	Constant deviation fault of the actuator
F2	Constant gain fault of the actuator
F3	Actuator stuck
F4	Actuator broken
F5	Constant deviation fault for both sensor and actuator
F6	Constant deviation fault of the sensor
F7	Constant gain fault of the sensor
F8	Sensor stuck
F9	Sensor broken
F10	Sensor and actuator are normal

## Data Availability

The data presented in this study are available on request from the corresponding author.
